# Multiparity, Brain Atrophy, and Cognitive Decline

**DOI:** 10.3389/fnagi.2020.00159

**Published:** 2020-06-03

**Authors:** Joon Hyung Jung, Ga Won Lee, Jun Ho Lee, Min Soo Byun, Dahyun Yi, So Yeon Jeon, Gi Jung Jung, Haejung Joung, Seong A Shin, Yu Kyeong Kim, Koung Mi Kang, Chul-Ho Sohn, Dong Young Lee

**Affiliations:** ^1^Department of Psychiatry, Seoul National University College of Medicine, Seoul, South Korea; ^2^Department of Neuropsychiatry, Seoul National University Hospital, Seoul, South Korea; ^3^Department of Neuropsychiatry, National Center for Mental Health, Seoul, South Korea; ^4^Department of Neuropsychiatry, Seoul National University Bundang Hospital, Seongnam, South Korea; ^5^Institute of Human Behavioral Medicine, Medical Research Center Seoul National University, Seoul, South Korea; ^6^Department of Neuropsychiatry, Chungnam National University Hospital, Daejeon, South Korea; ^7^Department of Nuclear Medicine, SMG-SNU Boramae Medical Center, Seoul, South Korea; ^8^Department of Radiology, Seoul National University Hospital, Seoul, South Korea

**Keywords:** Alzheimer’s disease, childbirth, multiparity, beta-amyloid, neurodegeneration, hippocampus

## Abstract

**Background:**

Multiparity – grand multiparity (i.e., five or more childbirths) in particular – has been reported to have an association with increased risk of Alzheimer’s disease (AD) dementia or related cognitive decline in women. However, the pathological links underlying this relationship are still unknown. This study was conducted to examine the relationships of multiparity with cerebral amyloid-beta (Aβ) deposition, brain atrophy, and white matter hyperintensities (WMHs).

**Methods:**

In this study, total of 237 older women with 148 cognitively normal and 89 mild cognitive impairment from the Korean Brain Aging Study for Early Diagnosis and Prediction of Alzheimer’s Disease (KBASE) were included. Participants underwent clinical and neuropsychological assessments in addition to ^11^C-labeled Pittsburgh Compound B positron emission tomography, and magnetic resonance imaging. The associations of parity with Aβ deposition, hippocampal volume, cortical volume, WMH volume and mini-mental status examination (MMSE) score were examined.

**Results:**

Participants with grand multiparity showed significantly reduced adjusted hippocampal volume, spatial pattern of atrophy for recognition of AD volume and spatial pattern of atrophy for recognition of brain aging volume even after controlling for potential confounders. Furthermore, MMSE score was also significantly lower in this group. In contrast, grand multiparity did not show any association with global Aβ retention, Aβ positivity rate, or WMH volume, regardless of covariates.

**Conclusion:**

Our findings suggest that grand multiparity contributes to cognitive decline or increased dementia risk in older women by aggravating amyloid-independent hippocampal or cortical atrophy.

## Introduction

The incidence and prevalence of Alzheimer’s disease (AD) dementia are higher in women than in men (Li and Singh, 2014). Gender-specific biological or psychosocial characteristics have been considered as potential factors underlying this difference (Snyder et al., 2016; Nebel et al., 2018). Pregnancy and childbirth are events specific to women and are associated with marked alterations in sex hormones, immunological factors, and lifestyle during pregnancy and the postpartum period (Cunningham et al., 2018). In particular, given the possible neuroprotective effects of estrogen, the marked changes in estrogen level associated with pregnancy and childbirth may impact the risk of AD dementia (Snyder et al., 2016; Nebel et al., 2018).

Several epidemiological studies reported that the number of childbirths or parity was related to increased risk of AD dementia or related cognitive decline (Colucci et al., 2006; Li et al., 2016; Jang et al., 2018). One recent study suggested that women with grand multiparity (i.e., five or more childbirths) exhibited a 1.7-fold higher risk of AD dementia than women with one to four parities (Jang et al., 2018). Grand multiparity was also linked to a 30% increase in the odds ratio (OR) for cognitive impairment in another study (Li et al., 2016). Similarly, three or more pregnancies were associated with higher frequency of AD dementia and earlier disease onset in a case-control study (Colucci et al., 2006). Taken together, the observations suggested that parity greater than a certain number, especially five or more, could increase the risk of AD dementia or related cognitive impairment. Grand multiparity has also been linked to increased risk of cardiovascular disease, cerebral infarction (Klingberg et al., 2017), and diabetes mellitus (Nicholson et al., 2006), which could influence risk of dementia or AD-related cognitive decline (Kalaria, 2000), as well as preeclampsia and other obstetric complications (Babinszki et al., 1999; Roman et al., 2004).

Despite the known relationships of multiparity, grand multiparity in particular, with AD dementia and related cognitive impairment, little is known about the neuropathological links underlying the relationship. While a postmortem brain study indicated that number of children was associated with neuritic plaque (Beeri et al., 2009), there have been no studies regarding the relationships between multiparity and brain pathologies or alterations in living human subjects.

Therefore, we investigated the relationship of grand multiparity with cerebral amyloid-beta (Aβ) deposition, brain atrophy, white matter hyperintensities (WMHs) and mini-mental status examination (MMSE) score in non-demented older women.

## Materials and Methods

### Participants

This study was part of the Korean Brain Aging Study for Early Diagnosis and Prediction of Alzheimer’s Disease (KBASE) (Byun et al., 2017b). This ongoing prospective study, which launched in 2014 was designed with the aims to reveal novel biomarkers for AD and to investigate contributions of various lifetime experiences to AD-related brain changes. A total of 237 non-demented older women [148 cognitively normal (CN) individuals and 89 with mild cognitive impairment (MCI)] between 65 and 90 years of age were included in the study. The CN subjects were not diagnosed with dementia or MCI and had a global Clinical Dementia Rating (CDR) score of 0. Subjects were classified as MCI if he or she met the following consensus criteria for amnestic MCI: (i) presence of memory complaint corroborated by an informant; (ii) objective memory impairment defined by the age-, education-, and gender-adjusted z-scores of less than −1.0 for at least one of the four episodic memory tests; (iii) preserved global cognitive function; (iv) essentially normal functions in instrumental daily activities; and, (v) without dementia (Winblad et al., 2004; Byun et al., 2017b). Episodic memory tests in the criterion (ii) included Word List Memory (immediate), Word List Recall (delayed), Word List Recognition, and Constructional Recall tests as part of the Korean version of the Consortium to Establish a Registry for Alzheimer’s Disease (CERAD-K) neuropsychological battery (Lee et al., 2004). The individuals with current and meaningful medical, psychiatric, or neurological disorders that could hinder mental functioning were excluded. Additionally, participants with the following conditions were excluded: presence of mental or physical conditions severe enough to interfere with brain imaging or clinical interview, no reliable informant, illiteracy, or being under the treatment with an investigational product or participation in a different clinical trial. The Institutional Review Board of Seoul National University Hospital and SNU-SMG Boramae Center in Seoul, Republic of Korea, approved the study, and written informed consent was obtained from the subjects.

### Clinical and Neuropsychological Assessments

Participants received standardized clinical and neuropsychological assessments by trained psychiatrists. This assessment followed the KBASE clinical assessment protocol, which included the CERAD-K assessment packet (Lee et al., 2002; Byun et al., 2017b). The KBASE neuropsychological assessments which incorporated the CERAD-K neuropsychological battery (Lee et al., 2004) were also administered to participants by trained psychometrists.

### Assessment of Reproductive History

Reproductive history was assessed through systematic interviews implemented by trained nurses. Items included age at menarche, age at menopause, number of pregnancies, and number of deliveries (parity). We divided the participants into two groups according to parity as follows: 0–4 parity and grand multiparity (i.e., five or more births) because grand multiparity has been repeatedly reported to be associated with AD dementia or cognitive decline as well as with higher rates of obstetric complications, cerebral infarction, cardiovascular diseases, and diabetes (Babinszki et al., 1999; Lawlor et al., 2003; Nicholson et al., 2006; Li et al., 2016; Keskin et al., 2017; Klingberg et al., 2017; Jang et al., 2018). While nulliparity is known to be associated with risk of several medical conditions and cognitive changes (Hanley et al., 2002; Lawlor et al., 2003; Mclay et al., 2003; Giubertoni et al., 2013), we did not classify nulliparity as an independent group because there were only two nulliparous subjects in our cohort.

### Assessment of Potential Confounders

Grand multiparity was previously reported to be associated with several socioeconomic conditions as well as health outcomes (Roman et al., 2004; Al-Shaikh et al., 2017; Klingberg et al., 2017), including low income, low or no formal education, and lack of employment (Roman et al., 2004; Al-Shaikh et al., 2017). As mentioned above, grand multiparity has also been linked to various vascular disorders (Nicholson et al., 2006; Klingberg et al., 2017). Therefore, all study participants were systematically evaluated for these potential confounders. Vascular risk factors (VRF), consisting of hypertension, diabetes mellitus, dyslipidemia, coronary heart disease, transient ischemic attack, and stroke, were assessed through systematic interviews. The VRF score (VRS) was calculated as the percentage of the number of VRF present (Decarli et al., 2004). Income level at early adulthood and lifetime occupation were also assessed through systematic interviews. Income level was classified into 3 groups according to the household income. The three groups were (a) low if below the minimum cost of living (MCL), (b) middle if below the twice the MCL, and (c) high if above the twice of MCL.^1^ The MCL was defined following the administrative rules announced in November 2012 by the Ministry of Health and Welfare, Republic of Korea. The minimum cost of living was 572,168 Korean Won for single-person households with addition of 286,840 Korean Won for each additional person. Lifetime occupation level was classified into 4 levels following the National Statistics Socio-Economic Classification (NS-SEC) (i.e., no lifetime occupation, routine and manual occupations, intermediate occupations, professional and managerial occupations) (Office for National Statistics, 2010).

### Measurement of Cerebral Amyloid Deposition

Using a 3.0T Biograph mMR (PET-MR) scanner (Siemens, Germany), we conducted 3D [^11^C] Pittsburg compound B (PiB)–positron emission tomography (PET), simultaneously with 3D T1-weighted MRI. Details on imaging acquisition and related preprocessing can be found elsewhere (Byun et al., 2017a). The PiB retention level was measured in the following 4 regions of interest (ROIs): the frontal, lateral parietal, posterior cingulate-precuneus, and lateral temporal regions. We applied the automated anatomical labeling algorithm and region combining method (Reiman et al., 2009) to define the ROIs and measure the PiB retention. We also integrated the 4 ROIs to define a global cortical ROI. The standardized uptake value ratio (SUVR) were calculated by diving the mean value for all voxels from each ROI with the mean cerebellar uptake value. A global Aβ retention value was similarly generated as a SUVR value for a global cortical ROI (Reiman et al., 2009). Subjects were defined as Aβ positive (Aβ +) if the SUVR value of at least one of the four ROIs was greater than 1.4 (Aβ +) (Reiman et al., 2009; Choe et al., 2014).

### Measurement of Brain Atrophy

We acquired the sagittal T1-weighted MRI using the aforementioned 3.0T PET-MR machine. Detailed methods for image acquisition and preprocessing have been described elsewhere (Lee et al., 2017). Obtained images were segmented automatically with FreeSurfer version 5.3.^2^ We calculated cortical regional volumes using the Desikan–Killiany atlas and for non-cortical regions, we used subcortical segmentation. The total hippocampal volume (HVt) was derived from adding the extracted hippocampal volumes of each hemisphere. Adjusted hippocampal volume (HVa) was calculated as a residual from a linear regression using the estimated total intracranial volume (ICV) and HVt of the young CN group of the cohort (ages between 25 and 54) as the reference group (Jack et al., 2014). Second, to determine the AD-type regional cortical atrophy pattern, we adopted the Spatial Patterns of Abnormality for Recognition of Early Alzheimer’s Disease (SPARE-AD) index, which was developed to quantify atrophy patterns associated with AD and reflects the volume of the following cortical ROIs: the hippocampus, inferior temporal gyrus, parahippocampal gyrus, posterior cingulate, precuneus, entorhinal cortex, and middle temporal gyrus (Fan et al., 2008). We calculated the sum of volumes of these ROIs, which was adjusted by ICV to yield the SPARE-AD volume. To examine the brain aging-related regional cortical degeneration pattern, we employed the Spatial Pattern of Atrophy for Recognition of BA (SPARE-BA) index (Habes et al., 2016b), which quantifies atrophy patterns associated with brain aging and reflects the volume of the following ROIs: the insular cortex, thalamus proper, cingulate cortex (anterior and middle), frontal, inferior parietal, and superior temporal gyrus, where significant volume differences were found between resilient aging and advanced brain aging (Habes et al., 2016b). The volumes of these ROIs were summed and adjusted by ICV to yield the SPARE-BA volume.

### Measurement of White Matter Hyperintensities

Cerebral WMH volumes were measured using fluid-attenuated inversion recovery (FLAIR) images obtained with the same MRI scanner. We adopted a previously validated automatic procedure (Tsai et al., 2014) with two modifications. First, a threshold value of 70 instead of 65 from the original reference was applied, which was more suitable for our data. Second, diffusion-weighted imaging was not used in the procedure as participants with acute cerebral infarcts were excluded.

### Statistical Analysis

To examine the association of parity groups [i.e., 0–4 parity and grand multiparity (independent variable)] with global Aβ retention, HVa, SPARE-AD volume, SPARE-BA volume, or WMH volume (dependent variables), we first performed multiple linear regression analyses. Two models were adopted. In Model 1, we controlled age, years of education, apolipoprotein E ε4 (APOE4) positivity, and cognitive status (i.e., CN vs. MCI) as covariates. In Model 2, we adjusted for the VRS, income level at early adulthood, level of lifetime occupation, age at menarche age, and age at menopause, in addition to the covariates included in Model 1. We also examined the association between MMSE score and parity using the covariates included in Model 2. In addition, multiple logistic regression analyses were performed to examine the association of parity group with Aβ positivity using the same covariate included in Models 1 and 2. All statistical analyses were conducted using SPSS software (version 23.0; SPSS Inc., Chicago, IL, United States), using two-tailed *p* < 0.05 to determine statistical significance. All statistical tests were repeated for sensitivity analyses, with exclusion of two nulliparous subjects.

## Results

### Characteristics of Participants

The demographic and clinical characteristics of the participants are summarized in Table 1. At baseline, the mean age of the 237 participants was 70.45 years (*SD* = 7.68). The 0–4 parity group had significantly higher education, younger age, higher MMSE score, and greater HVa, SPARE-AD, and SPARE-BA volume than the grand multiparity group. There were no differences with regard to income level at early adulthood, level of lifetime occupation, age at menarche or age at menopause between the two groups. Additionally, APOE4 positivity, cognitive status, global Aβ deposition, Aβ positivity, and WMH volume were also comparable between the two groups.

**TABLE 1:** Characteristics of the participants.

**Characteristics**	**Overall (*n* = 237)**	**Parity (number of deliveries)**		**t or χ^2^**	***p*-value**
		**0–4 (*n* = 210)**	**5 or more (*n* = 27)**		
Age, years	70.45 ± 7.68	69.46 ± 7.37	78.15 ± 5.42	–7.487	<0.001
Education, years	9.71 ± 4.71	10.10 ± 4.59	6.67 ± 4.66	3.655	<0.001
ApoE ε4 positivity n (%)	59 (24.9)	54 (25.7)	5 (18.5)	0.663	0.416
Cognitive status				0.132	0.716
CN, n (%)	148 (62.4)	132 (62.9)	16 (59.3)		
MCI, n (%)	89 (37.4)	78 (37.1)	11 (40.7)		
MMSE	24.93 ± 3.55	25.21 ± 3.29	22.70 ± 4.62	2.734	0.010
Global Aβ deposition (SUVR)^†^	1.297 ± 0.368	1.30 ± 0.37	1.29 ± 0.36	0.072	0.942
PiB positivity, n (%)^†^	50 (21.1)	43 (20.5)	7 (26.9)	0.576	0.448
Adjusted hippocampal volume^††^	−919.7 ± 1061.9	−823.8 ± 1033.3	−1651.5 ± 1007.3	3.925	<0.001
SPARE-AD volume^††^	0.054 ± 0.005	0.055 ± 0.005	0.051 ± 0.003	3.485	0.001
SPARE-BA volume^††^	0.126 ± 0.009	0.126 ± 0.009	0.120 ± 0.008	3.621	<0.001
White matter hyper-intensity^‡^(cm^3^)	5.75 ± 5.48	5.52 ± 5.23	7.66 ± 7.01	–1.422	0.167
Number of pregnancies	4.85 ± 2.61	4.50 ± 2.09	7.59 ± 4.27	–6.235	<0.001
Number of deliveries (parity)	3.02 ± 1.22	2.71 ± 0.88	5.44 ± 0.64	–15.548	<0.001
0 (n, %)	2 (0.8)				
1–2 (n, %)	85 (35.9)				
3–4 (n, %)	123 (51.9)				
≥5 (n, %)	27 (11.4)				
Age at menarche, years	15.80 ± 1.95	15.72 ± 2.00	16.42 ± 1.45	–1.733	0.084
Age at menopause, years	50.50 ± 5.17	50.56 ± 5.14	50.07 ± 5.46	0.460	0.646
Income level at early adulthood				0.456	0.820
Low	53 (22.4)	47 (22.4)	6 (22.2)		
Middle	134 (56.5)	120 (57.1)	14 (51.9)		
High	50 (21.1)	43 (20.5)	7 (25.9)		
Level of lifetime occupation				6.454	0.084
No	77 (32.6)	68 (32.4)	9 (11.7)		
Routine and manual	43 (18.2)	36 (17.1)	7 (26.9)		
Intermediate	82 (34.7)	79 (37.1)	4 (15.4)		
Managerial and professional	34 (14.4)	28 (13.3)	6 (23.1)		
VRS	19.06 ± 16.21	18.57 ± 15.78	22.84 ± 19.14	–1.290	0.198

*Data are presented as mean ± SD or N(%). For comparison of demographics between the 0 and 4 parity and 5 or more parity groups, independent T-test was used for continuous variables and either Chi-square test or Fischer’s exact test was used for categorical variables. ApoE, apolipoprotein E; CN, cognitively normal; MCI, mild cognitive impairment; MMSE, mini-mental state examination; Aβ, amyloid beta; SUVR, standardized uptake value ratio; PiB, Pittsburgh compound B; SPARE-AD, spatial patterns of abnormality for recognition of early AD; SPARE-BA, spatial pattern of atrophy for recognition of brain aging; VRS, vascular risk factors score. ^†^n = 236, ^††^n = 233, ^‡^n = 210.*

### Parity and Brain Changes

**FIGURE 1:**
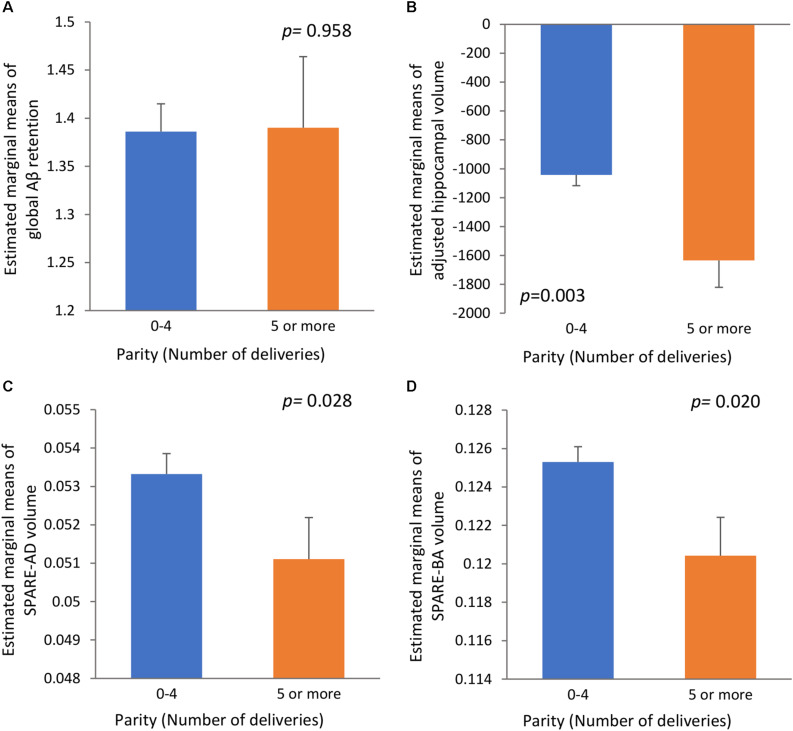
The relationships **(A)** between parity and global Aβ retention, **(B)** between parity and adjusted hippocampal volume, **(C)** between parity and SPARE-AD volume, and **(D)** between parity and SPARE-BA volume with standard errors. Adjusted for age, education, APOE4 positivity, cognitive status, VRS, income level at early adulthood, level of lifetime occupation, age at menarche age and age at menopause. Aβ, β-amyloid; SPARE-AD, spatial patterns of abnormality for recognition of early AD; SPARE-BA, spatial pattern of atrophy for recognition of brain aging; APOE4, apolipoprotein E ε4; VRS, vascular risk factors score.

We found no significant association between parity group and global Aβ deposition, regardless of the statistical models used (Table 2 and Figure 1A). Similarly, we observed no association between parity group and Aβ positivity (Table 3). In contrast, the grand multiparity group exhibited significantly smaller HVa, SPARE-AD volume and SPARE-BA volume than the 0–4 parity group in both Model 1 and 2 (Table 2 and Figures 1B–D). The association between parity group and WMH volume was not significant (Table 2), regardless of the model used.

**TABLE 2:** Relationship of grand multiparity with global Aβ retention, adjusted hippocampal volume, SPARE-AD volume, SPARE-BA volume, and WMH.

	**Model 1^†^**				**Model 2^††^**			
	***B***	***SE***	***t***	***P***	***B***	**SE**	***t***	***P***
Global Aβ retention	0.018	0.071	0.258	0.796	0.004	0.077	0.053	0.958
HVa	−418.1	185.8	–2.251	0.025	−591.2	194.2	–3.044	0.003
SPARE-AD volume	−0.002	0.001	–2.069	0.040	−0.002	0.001	–2.209	0.028
SPARE-BA volume	−0.004	0.002	–2.338	0.020	−0.005	0.002	–2.340	0.020
WMH	0.849	1.274	0.667	0.506	0.958	1.380	0.694	0.489

*^†^Adjusted for age, education, APOE4 positivity, cognitive status. ^††^Adjusted for age, education, APOE4 positivity, cognitive status, VRS, income level at early adulthood, level of lifetime occupation, age at menarche age and age at menopause. Aβ, amyloid beta; HVa, adjusted hippocampal volume; SPARE-AD, spatial patterns of abnormality for recognition of early AD; SPARE-BA, spatial pattern of atrophy for recognition of brain aging; WMH, White matter hyperintensities; APOE4, apolipoprotein E ε4; VRS, vascular risk factors score.*

**TABLE 3:** Results of the multiple logistic regression analyses of grand multiparity with Aβ positivity.

	**Model 1^†^**		**Model 2^††^**	
	**OR (95% CI)**	***P***	**OR (95% CI)**	***P***
Aβ positivity	1.774 (0.583–5.398)	0.313	1.453 (0.384–5.492)	0.582

*^†^Adjusted for age, education, APOE4 positivity, cognitive status. ^††^Adjusted for age, education, APOE4 positivity, cognitive status, VRS, income level at early adulthood, level of lifetime occupation, age at menarche age and age at menopause. OR, Odds ratio; CI, Confidence interval; Aβ, amyloid beta; APOE4, apolipoprotein E ε4, GDS, geriatric depression scale; VRS, vascular risk factors score.*

### Parity and Cognition

With regard to cognition, the grand multiparity group showed lower MMSE scores than the 0–4 parity group (Model A in Table 4). When we additionally controlled for HVa, SPARE-AD volume and SPARE-BA volume (Model B in Table 4), the relationship between parity and MMSE score was no longer significant indicating the mediating effect of HVa, SPARE-BA volume and SPARE-AD volume.

**TABLE 4:** Relationship of grand multiparity with MMSE score.

	**MMSE**			
	***B***	***SE***	***t***	***P***
Model A^a^	−1.381	0.595	−2.321	0.021
Model B^b^	−1.102	0.607	−1.815	0.071

*^*a*^Adjusted for age, education, APOE4 positivity, cognitive status, VRS, income level at early adulthood, level of lifetime occupation, age at menarche age and age at menopause. ^*b*^Adjusted for the covariates of Model A plus HVa, SPARE-AD volume and SPARE-BA volume. MMSE, mini-mental status examination; APOE4, apolipoprotein E ε4; VRS, vascular risk factors score; HVa, adjusted hippocampal volume; SPARE-AD, spatial patterns of abnormality for recognition of early AD; SPARE-BA, spatial pattern of atrophy for recognition of brain aging.*

### Sensitivity Analyses

The overall results were nearly identical to those described above when we repeated the same analyses after excluding the two nulliparous subjects as sensitivity analyses.

## Discussion

We found that grand multiparity was associated with reduced brain volume, particularly hippocampal volume, but not with Aβ pathology or WMHs, in non-demented older women. While grand multiparity was also related to a lower MMSE score, the relationship disappeared when adjusted for brain atrophy. As far as we aware, this is one of the first study to examine the association of parity with *in vivo* brain changes in women in later life.

In the present study, grand multiparity showed no significant association with global Aβ deposition or Aβ positivity. The null association between grand multiparity and *in vivo* Aβ pathology observed here was consistent with the results of a previous preclinical study that indicated no differences in Aβ pathologies between nulliparous and multiparous female mice (Ziegler-Waldkirch et al., 2018). However, in contrast to our results, a postmortem human brain study with a small sample size (*n* = 73) indicated a positive association between number of children and neuritic plaque in women (Beeri et al., 2009). As pregnancy and childbirth are complex phenomena that incorporate both marked biological and social changes, potential factors that may confound the relationship between parity and brain pathology should be well controlled. Nevertheless, the postmortem brain study mentioned above did not control for such potential confounders. In addition to the advantage of measuring *in vivo* brain changes, our study had a larger sample size (*n* = 237), which made it possible to adjust various potential confounders as covariates.

In contrast to amyloid pathology, hippocampal atrophy was closely related to grand multiparity.

While exact mechanisms driving the atrophy remain to be fully clarified, a couple of possible explanations could be made. Repeated stress associated with multiple childbirths may explain the relationship. Women experience extreme physical and emotional stress during childbirth and up to 16% of women exhibit clinically important PTSD symptoms (Horsch and Ayers, 2016; Dekel et al., 2017). Additionally, raising multiple children may lead to chronic stress and this could be more prominent when raising children with behavioral problems or illnesses (Barroso et al., 2018; Holly et al., 2019). Hippocampal atrophy is well known for its association with stress and the hypopituitary-pituitary-adrenal axis hyperactivity (Lupien et al., 2009). Repeated or chronic stress increase glucocorticoid and induce hippocampal atrophy in both human and animal model (Gianaros et al., 2007; Lupien et al., 2009).

Another possible explanation is that marked changes in sex hormone during pregnancy and the postpartum period, especially changes in estrogen level (Prange-Kiel and Rune, 2006; Bridges, 2016), may cause hippocampal atrophy. The estrogen level is known to show an inverted U-shaped relationship with hippocampal volume and memory, suggesting that not only low estrogen but also high estrogen level may be associated with cognitive decline (Bean et al., 2014; Bayer et al., 2018). During pregnancy, estradiol levels can reach 200 times the preconception levels by week 20 and can be 1000 times higher than preconception levels prior to childbirth (Shansky, 2015). At parturition, with the expulsion of the placenta, there are rapid decreases in steroid and peptide hormone levels, and women are in a hypogonadal state for up to 180 days as lactation is linked to anovulation and a hypoestrogenic state (Shansky, 2015; Cunningham et al., 2018). Furthermore, even after the postpartum period, estrogen level was reported to be 22% lower in parous than in nulliparous women (Bernstein et al., 1985). In summary, both supraphysiological estrogen level during pregnancy and decreased estrogen level during and afterward the postpartum period could contribute to hippocampal atrophy.

Current estradiol levels were not different between the 0–4 parity and grand multiparity groups (data not shown) in our study. In addition, our previous study showed that the current level of estradiol was not associated with hippocampal volume (Lee et al., 2017). Taken together, these observations suggested that marked fluctuations in previous estrogen level associated with repeated pregnancy and childbirth have a large negative impact on the hippocampus, regardless of the current estrogen level.

In addition to hippocampal atrophy, diffuse cortical brain atrophy was also related to grand multiparity. Both SPARE-BA and SPARE-AD volumes were significantly lower in the grand multiparity group than in the 0–4 parity group. These findings suggest that grand multiparity-associated brain atrophy is not specific to the AD-related pathological process and also closely related to accelerated brain aging. This is also consistent with the null association between grand multiparity and Aβ deposition observed in the present study. It is not clear how grand multiparity induces changes in these cortical regions. Increased levels of estrogen and/or cortisol during pregnancy could also play roles in changes in these regions as well as the hippocampus (Geerlings et al., 2015; Shansky, 2015). The association of grand multiparity with increased risk of vascular disease (Hanley et al., 2002; Nicholson et al., 2006; Giubertoni et al., 2013; Klingberg et al., 2017) may also explain brain impairment, because both SPARE-BA and SPARE-AD are vulnerable to vascular risks and WMH (Habes et al., 2016a, b). In the present study, however, WMH was not associated with grand multiparity. Moreover, the association between parity and cortical atrophy remained significant even after adjusting for VRS.

In addition, grand multiparity was related to lower MMSE score, which was consistent with previous reports showing relations between grand multiparity and increased risk of AD dementia and cognitive decline or earlier onset of dementia (Colucci et al., 2006; Li et al., 2016; Jang et al., 2018). The relationship between parity and MMSE score disappeared when we controlled for the neurodegeneration markers (i.e., HVa, SPARE-AD volume, and SPARE-BA volume), indicating that the influence of grand multiparity on cognitive decline and increased risk of dementia is mediated by accelerated atrophy or neurodegeneration of these brain regions.

This study had some limitations. First, as this was a cross-sectional study, it is difficult to infer causal relationships from the findings, and further longitudinal studies are needed to demonstrate causal links. Second, reproductive history was assessed by self-report and retrospective recall, and there was therefore a risk of recall bias. However, we obtained additional information or re-confirmed the reproductive history of the participants by interviews with reliable informants. In most cases, the numbers of children or parity recalled by the participants were not different from the reports of these informants. Finally, although nulliparity may be separately associated with the risk of cognitive decline, we could not analyze its impact because the number of nulliparous subjects included in the study was too small.

## Conclusion

The findings of the present study suggest that grand multiparity contributes to cognitive decline or increased dementia risk in older women by aggravating amyloid-independent hippocampal or cortical atrophy, while exact mechanisms driving such atrophy remain to be fully clarified. In regard of brain health and dementia prevention, this may be especially important for women in developing countries where the number of childbirths is still high.

## Data Availability Statement

The datasets generated for this study are available on request to the corresponding author.

## Ethics Statement

The studies involving human participants were reviewed and approved by Institutional Review Board of Seoul National University Hospital and SNU-SMG Boramae Center in Seoul, South Korea. The patients/participants provided their written informed consent to participate in this study.

## Author Contributions

JJ, GL, and DL contributed to conception and design of the study, drafting the manuscript, and preparing the figures. All authors contributed to data acquisition and analysis.

## Conflict of Interest

The authors declare that the research was conducted in the absence of any commercial or financial relationships that could be construed as a potential conflict of interest.

## Footnotes

1 http://www.law.go.kr

2 http://surfer.nmr.mgh.harvard.edu/

## Abbreviations

AD: Alzheimer’s disease
ApoE4: apolipoprotein E ε 4
A β: Amyloid-beta
CDR: Clinical Dementia Rating
CERAD-K: Korean version of the Consortium to Establish a Registry for Alzheimer’s Disease Assessment Packet
CN: cognitively normal
HVa: adjusted hippocampal volume
HVt: total hippocampal volume
ICV: intracranial volume
KBASE: Korean Brain Aging Study for Early Diagnosis and Prediction of Alzheimer’s Disease
MCI: mild cognitive impairment
MCL: minimum cost of living
NS-SEC: National Statistics Socio-Economic Classification
PET: positron emission tomography
PiB: Pittsburg compound B
SPARE-AD: Spatial Patterns of Abnormality for Recognition of Early Alzheimer’s Disease
SPARE-BA: Spatial Pattern of Atrophy for Recognition of brain aging
SUVR: standardized uptake value ratio
VRF: Vascular risk factors
VRS: Vascular risk factors score
WMH: white matter hyperintensity.
